# Design of price incentives for adjunct policy goals in formula funding for hospitals and health services

**DOI:** 10.1186/1472-6963-8-72

**Published:** 2008-04-03

**Authors:** Stephen J Duckett

**Affiliations:** 1 Reform and Development Division, Queensland Health and Australian Centre for Economic Research on Health, University of Queensland, Australia

## Abstract

**Background:**

Hospital policy involves multiple objectives: efficiency of service delivery, pursuit of high quality care, promoting access. Funding policy based on hospital casemix has traditionally been considered to be only about promoting efficiency.

**Discussion:**

Formula-based funding policy can be (and has been) used to pursue a range of policy objectives, not only efficiency. These are termed 'adjunct' goals. Strategies to incorporate adjunct goals into funding design must, implicitly or explicitly, address key decision choices outlined in this paper.

**Summary:**

Policy must be clear and explicit about the behaviour to be rewarded; incentives must be designed so that all facilities with an opportunity to improve have an opportunity to benefit; the reward structure is stable and meaningful; and the funder monitors performance and gaming.

## Background

Over the last twenty-five years, hospital funding systems have evolved to reduce idiosyncratic and historical ways of funding, replacing these methods with formulae or market based systems, such as casemix funding, to promote technical efficiency, minimising cost per patient treated [1].

But emphasising technical efficiency is not the only objective of health funding policy: in capped funding environments there needs to be a parallel focus on access, and in all environments, a focus on quality. With the singular exception of the equity-oriented 'disproportionate share' incentives in the US Medicare prospective payment design [2], in early formula funding implementations policy instruments to achieve "adjunct" policy goals of access and quality were regulatory (or hierarchical) in nature. As the ability to measure performance in domains relevant to adjunct goals has improved, control mechanisms shifted from hierarchical to market-like mechanisms [3,4] and a literature on the effectiveness of incentives developed [5,6]. The 'equal compensation principle', from the economics literature, postulates that when employees (or subsidiary organisational units) are assigned multiple tasks/goals, compensation design must ensure that the marginal rate of return to the employee (unit) for all tasks is equal [7]. In line with this, contemporary formula funding arrangements are increasingly using market-like incentives for adjunct goals (for an argument favouring mixed systems of payment when multiple objectives are being pursued see Eggleston [8]). The United States Institute of Medicine has recommended expansion of pay for performance [9] and providers expect that such incentive programs will grow in scope in the future [10]. Berg et al has extended this approach to vary *consumer *co-payments to take account of adjunct goals, specifically quality [11].

An emphasis on adjunct goals also emerged because of recognition of weakness in measures of hospital product. The standard measure of inpatient activity, a treated patient, incorporates an implicit assumption about homogeneity of quality of care and other attributes. To the extent these attributes are unmeasured (or unmeasurable) the purchaser (such as primary care trusts in the United Kingdom or an Area Health Authority), or principal, is exposed to the risks involved in asymmetry of information with agents: moral hazard (such as saving money by skimping on quality because it is unmeasured) and adverse selection (providers selecting patients likely to be less costly). Optimal contracts in these situations should involve improved signalling about desirable behaviour [12] and increased use of adjunct incentives is one approach to this.

There are two broad approaches to funding health services: casemix and population. Population-based funding is designed to improve efficiency by promoting appropriate location of care and investment in prevention, but it also promotes a quality-oriented adjunct goal of improving care continuity through service integration [13]. Apart from that design feature, adjunct incentives are more commonly seen in casemix funding systems compared to population-based funding systems, partly because of casemix's stronger emphasis on incentives on providers, so this paper focuses on incentives in the context of casemix funding.

Market-like incentives for adjunct goals are designed to strengthen the alignment of the goals of peripheral units (areas, regions, hospitals) and those of the centre. This paper identifies potential adjunct incentives and outlines principles associated with the design of incentives to be incorporated into casemix funding policy. It is primarily focused on incentives relating to the principal "product stream" in hospital funding: patient treatment. It does not address incentives associated with other key product streams such as clinical education or research. Nor does it address cost-related adjusters (such as for specific equity groups), which may provide effective incentives for access for these groups.

## Discussion

### Potential Instruments

A range of potential market-like instruments can be used to create adjunct incentives. Instruments can be characterised on a number of key dimensions:

• Goal, target or domain

• Nature of the mechanism or instrument (continuous or threshold based)

• Uncapped vs. capped

• Competitive vs. non-competitive

• Positive or negative

• Timing

The principal categorisation of adjunct incentives is the *goal, target or domain *that is the object of the incentive. Adjunct incentives have been designed across a range of domains including quality of care, access, prevention and coding quality.

A key role for adjunct incentives is to counteract perverse or unintended consequences of the funding formula. Some funding arrangements may effectively reward poor quality, for example, encouraging over utilisation or unnecessary diagnostic testing [14]. Under casemix funding the inherent efficiency incentives may lead hospitals to seek to achieve improved efficiency, narrowly defined, by reducing quality of care. In the US Medicare implementation of prospective payment, this perverse incentive was redressed through monitoring by Professional Standards Review Organisations [2]; in the Victorian (Australia) implementation, a monetary incentive was used to reward hospital accreditation, supplemented by a regulatory approach, monitoring of readmissions [15].

The quality domain has attracted the greatest interest of all potential areas for introducing market-based adjunct incentives, complementing other quality-related strategies such as selective contracting and use of report cards [16,17]. There has been a significant increase in interest in incentives for provision of higher quality of care in the United States in recent years, with interest across all phases of improved reporting ('pay for reporting'), pay for participation in quality improvement initiatives, and 'Pay for Performance' or 'P4P' [18]. Although P4P risks being another 'unicorn' in terms of health policy, much talked about, never seen [19], several quality incentives have been developed, for example, including a bonus payment for treatments that adhere to particular care paths or for care meeting specified process indicators [20]. Similar initiatives have been implemented in Nicaragua and Costa Rica [21] and are under consideration in Canada [22]. Averill et al have recently proposed to incorporate a quality adjustment directly into the price paid by removing the effect of complications (as distinct from co-morbidities present on admission) from casemix assignment [23]. Gosfield has proposed a new payment approach based on funding according to care paths, with bonuses for efficiency and consumer satisfaction [24]. Although the empirical support for some of the initiatives is weak [25,26], hospitals have been shown to respond to these incentives with process or structural changes [27-29] and they can strengthen the internal 'business case' for implementing patient safety initiatives [30]. An important study by Lindenauer et al showed a statistically significant improvement in quality measures compared to controls for quite modest levels of incentives at the margin: 1–2% depending on performance [31].

In capped funding systems, incentives for hospitals to address priority access goals are also important. Here incentives on area services to redress a perverse incentive to under-provide may be required. The Victorian implementation of casemix funding incorporated a strong incentive for hospitals to ensure that urgent elective surgery cases were treated within a clinically acceptable time frame [32]. Subsequent adjunct incentives were introduced in Victoria to place a parallel incentive for hospital emergency department performance [33,34].

Prospective payment arrangements generally incorporate measures to ensure appropriate coding quality such as audits, with penalties for poor coding performance, and incentives to ensure coding timeliness. It is also possible to contemplate incentives on area health services or hospitals in terms of prevention, particularly through discounted payments for admissions which might be better treated in a primary care setting and incentives for appropriateness of admission, through discounts for excess admissions in procedures exhibiting high variability. Process measures of good preventive care have also been used in the design of incentives for managed care organisations [25] and general practice [35].

Table 1 shows indicators that can be used as a basis for adjunct incentives in a range of domains.

**Table 1:** DOMAINS OF PERFORMANCE AND POTENTIAL INDICATORS

**DOMAIN**	**INDICATOR**	**POSSIBLE INCENTIVE DESIGN**
QUALITY	• Clinical indicators e.g. % adherence to specific treatment for specific disease	• Incremental payment where evidence of specific indicator
	• Adherence to (any) endorsed care path	• Increment for adherence to care path
	• Provision of data to allow clinical benchmarking	• Payment for provision of data
	• Achievement of hospital accreditation	• Bonus for accreditation
	• Complications which arise during course of treatment (such as adverse events)	• Remove *complications *(which occur after the patient was admitted, contrasting with *comorbidities *which were present on admission) from the definition of DRG and hence determination of casemix payment
	• Score on consumer satisfaction questionnaire	• Incremental payment
	• Appropriateness of care such as measured by agreed instrument	• Discount payment for cases which do not meet appropriateness of admission criteria as they are of less 'value' to purchaser
	• Propensity to admit conditions that exhibit high geographic variation such as carpal tunnel operations.	• Reduce "profitability" of these cases by discounted payment for admission of high variability conditions

ACCESS	• Elective surgery waiting times	• Discount/penalties for high percent or number of patients waiting in excess of threshold time
		• Premium paid for patients treated within acceptable timeframe (or penalty for revenue).
		• Additional payments (or access to other types of additional funding arrangements) if negotiated target reduction in long wait patients achieved.
	• Hospital emergency service times to treatment (by triage category)	• Penalties for failure to achieve threshold treatment time goals
	• Long stays in hospital emergency service	• Penalties for number of patients denied timely admission to ward.

PREVENTION	• Avoidable hospital admissions	• Discounted payment for avoidable admissions
	• Avoidable mortality	• Penalty in population funding formula for excess avoidable mortality

CODING QUALITY AND TIMELINESS	• Timeliness	• Zero payment for submission of data outside specific timeframes
	• Incidence of "error" DRGs	• Discounted payment for 'error' DRG codes.
	• Coding error as measured by audit	• Penalty for upcoding (eg. double deduction where overcoding found).

With multiple goals comes the prospect of goals conflict e.g. is there a trade-off between quality and access? Incentive design can explicitly deal with such goal trade-off by incorporating multiple measures to determine incentive payments. The relative size of any bonus or penalty (or the relative weighting in any aggregate measure of performance) implicitly reveals the preferences of purchasers/funders as to the relative importance of the different goals. As noted below, performance thresholds for access to incentives can also be used to address multiple goals.

A second broad characteristic of market-like adjunct incentives relates to the design of the incentive in terms of the *mechanism or instrument*. Here there are two broad approaches to instrument design with both having advantages [36]. The first involves a positive relationship to the potential indicator, examples include an incremental adjustment to the prospective payment price for quality or a penalty related to each patient waiting longer than the clinically acceptable waiting time. The alternate approach is based on thresholds: a particular level of performance is required before the reward or penalty applies. For example, in the original design of casemix funding in Victoria, access to payment for additional activity was conditional on a particular level of waiting time performance for urgent elective surgery patients. The contemporary Victorian design provides for penalties (and bonuses) relating to particular thresholds of activity (eg. a hospital being on ambulance bypass more than 3% of the time). Thresholds are particularly appropriate when there are designated minimum performance standards. Nolan and Berwick have argued that dichotomous or 'all-or-none' measures of performance send more powerful signals to focus quality improvement efforts [37]. However, because improvements in performance below the threshold are unrewarded, the worst performers (who are least likely to meet the threshold) may make no effort to improve at all.

As with most aspects of incentive design, the two approaches described here are not alternatives and can co-exist. Thresholds could limit access to a particular incentive (payment quantum) or achieving the threshold could be a condition precedent for accessing another incentive system (such as additional payment for additional activity, an approach used in the initial Victorian payment design [15]). In the latter case, the threshold criteria need not be in the same domain as the continuous incentive for example, quality and safety performance could be a threshold criterion for accessing an additional volume-related payment for activity.

As with the funding arrangement as a whole, adjunct incentives can either be *capped or uncapped*. There is again a trade-off here: total expenditure potentially to be met by the funder/purchaser versus strength of an incentive in terms of the potential revenue to facilities/providers. Even in a capped funding system, uncapped incentives might be acceptable depending on the funder/purchaser's assessment of the risk of providers achieving higher levels of performance and thus earning higher levels of reward, and the (relative) priority the funder/purchaser ascribes to that domain.

Adjunct incentives can be structured in ways that are *competitive or non-competitive*. In terms of competitive arrangements, a bonus pool could be set aside for a particular region or State and an individual hospital's share of that pool would depend on its performance relative to its peers. The initial Victorian application was of this kind in part because of the need to cap funding [15], as is the scheme in Rochester, New York [38]. The alternative would be for a bonus pool to be specified for a particular hospital and bonus payments to the hospital would be independent of the performance of other hospitals. A competitive bonus pool has a number of weaknesses: lack of certainty to providers (as their bonus depends not only on their *relative *not simply actual performance and, for the funder/purchaser, the risk that small improvements in performance might lead to very large payments, depending on overall system performance.

Incentives can be phrased and effected *positively or negatively*: structured as 'bonuses' or incremental payments or 'penalties', reduction in funding which would otherwise be available to the entity. Positively phrased incentives ('bonuses') will obviously be better received than negatively phrased ones. But potential bonuses may become incorporated in funding expectations and, even though officially phrased positively, failure to achieve the bonus may still be perceived as a penalty. Both bonuses and penalties can have important motivational effects, especially in environments (such as competitive markets) where providers place a premium on reputation. But both bonuses and penalties might also demotivate, in the first instance allowing excellent providers to 'rest on their laurels' and in the alternate case, inducing despondency in poor performers and exit from the incentive system. If penalties involve true reductions in funding for poor performance, this might induce a vicious cycle where providers cannot generate discretionary funds (even on a once-off basis) to implement necessary changes in processes to improve their performance.

Finally, consideration needs to be given to the *timing *of the incentive and payment continuity. Incentives can be paid continuously (a premium or reduction paid for every patient which meets criteria) or periodically. In the event of periodic payment, the incentive should become available to the provider at a time proximate to the performance being recognised (quarterly, half yearly, annually), with more frequent payments needing to balance the risk of confusing seasonality or chance with true underlying change in performance coupled with the added administrative burden of frequent payments on the one hand with the benefits of more frequent feedback on performance and inducing desired changes in performance more frequently on the other. Frequent payments will also inevitably reduce each payment aliquot, potentially reducing the strength of the incentive.

Obviously programs with different design features will distribute rewards (and penalties) differently even if they target the same attributes [39]. The design of an adjunct incentive policy needs to address, explicitly or implicitly, all of these dimensions with the appropriate choices being influenced by history, culture, information systems, management capacity and other contextual factors specific to the health system or services for which the purchaser/funder is responsible. Design processes are also important. An implementation in Maine, for example, had two separate groups involved in incentive design: one identifying and agreeing on the indicators to be used and a second designing the incentive structure associated with the agreed indicators [40].

### Principles for use of market-like incentives

There are extensive literatures from a range of disciplines on governance and design of incentives to influence individual or organisational decision-making [13,17,41]. Although some research questions remain [42] and there is still need for conceptual development [15], these literatures can be used to distil a number of core principles relevant to the design of market-like incentives to achieve adjunct policy goals [43]. The core principles of economics provide the first insight: incentives apply at the margin of behaviour and relatively small increments/decrements in funding can be used to affect performance. A rational manager with perfect information operating in a perfect market (i.e. the standard assumptions of Economics 101) will improve quality of services up to the point where the marginal gain from improving quality equals the marginal return [44]. The impact of the marginal incentives is thus effectively to change the returns from quality for providers who meet the criteria. Depending on a provider's position on the marginal cost curve and whether or not they operate in a competitive environment, different incentive designs may not impact on the provider, or at least not impact on them in a way that causes them to respond to the incentive. This theoretical analysis may explain some of the equivocal and contradictory results from evaluations of pay for performance programs in practice [45].

Several sub-fields of economics are also relevant [6]. Transaction cost economics has developed to inform whether markets (or market-like incentives) versus hierarchies or regulation should be used to govern transactions ([4,45,46]. Similar principles apply to the choice between market-like incentives and regulatory instruments to achieve adjunct policy goals (for an application of the transaction cost economics framework in the health sector see Ashton [47]). A second stream in the economics literature, relating to the theory of contracts, can also inform incentive design [48,49]. Here it is recognised that many contracts are 'incomplete' in the sense that all contingencies and potential responses cannot be specified in advance and theory informs how, in these circumstances, contracts must be written to ensure an appropriate distribution of risk.

A key issue in incentive design relates to the unit that is to be the target of incentives, and the first question here is whether the incentive is to be focussed on an individual manager or clinician or on an organisational unit [14,50], and whether incentives are to be directly financial ('high powered') or reward indirectly, through promotion opportunities ('low powered'). This choice is not strictly about alternatives, as performance appraisal/individual bonuses aligned with the adjunct incentives can be used in concert with overall payment system design (for a recent review of individual incentives see [51], for economics perspectives see [52-54]). Thus the performance contract for an individual manager could have an element specifically related to achievement of organisational rewards flowing from a wider adjunct incentive arrangement.

Incentives on the organisation as a whole can also be passed onto the appropriate internal structures, which are accountable for relevant aspects of performance (eg, the management group or medical staff [55]). The choice of organisational level to be the target of incentives will be in part determined by the balance of centralisation and decentralisation in the overall system design and will depend on the degree of autonomy to be allotted to sub-units. Greater levels of autonomy can be supported if financial incentives promote alignment of goals of sub-units with the overall organisation. Hospitals are characterised by a high degree of interaction – sequential or pooled interdependence to use Thompson's terms [56] – in turn suggesting group or team performance needs to be the focus of rewards and incentives. This choice is in part a matter of manager preference and thus an option is to place incentives on an organisation and allow the organisation management to determine intra-organisational distribution policies.

As discussed earlier, health policy involves inherent tradeoffs between multiple objectives and competing demands (e.g. elective surgery versus emergency care). The choice of organisational level for funding incentives needs to take account of where these tensions are to be resolved, further down the organisational hierarchy or higher up depending on the centralising tendency of the organisation. Whether incentives are targeted at individuals or organisations, the incentives need to be aligned to professional values [57]: as the 1990s experience with managed care in the United States suggests, financial incentives which are antithetical to professional values create a backlash and do not work in the long term. Provider attitude to salience, clinical relevance and other dimensions of incentive design can be robustly measured before (and/or after) the introduction of an incentive payment [58].

There is also choice of the organisational level for targets in multi-hospital systems [22]. Although the international trend is for corporatised hospitals, most Australian states have now adopted integrated health system structures [59], most Canadian provinces have also regionalised and regionalisation is a common trend in many European nations. Although they function as regional intermediate structures between the central office and the hospital, these "meso" structures are often integral parts of the health authority/department and are not separately incorporated entities. Do they behave like 'principals' or 'agents'? A key choice in the design of incentives is thus whether the intermediate entity is seen as part of the central office and not subject to adjunct incentives (with adjunct goals being driven, for example, through personnel appraisal) or whether system incentives will apply to area or regional budgets. The literature offers little guidance for this choice.

The fundamental principle about which there is guidance derives from application of what works in terms of conditioning, behavioural design and design of indicators. The psychology literature has identified a number of key elements that can be synthesised into an emphasis on a clear link between the behaviour and the reward. A condition precedent of this is that the desired behaviour must be measurable; that is there must be a clear and measurable indicator that validly measures the desired behaviour or outcomes and the associated target [22,60], in the health sector this also implies that all indicators have been subject to appropriate risk or casemix adjustment. There must be room for improvement [61], but the relevant target must be achievable: if the target set is too much of a "stretch goal", then the hospital or area may assume that there is no point attempting to reach the target and the whole point of the incentive is vitiated: a principle described as the 'rule' having 'some expected usefulness' [62]. The obverse risk to only rewarding extraordinary performance change is also relevant: the incentive design should eschew rewards for trivial change and should only reward policy-relevant changes in performance, avoiding the complexity inherent in managing a system of rewards for marginal changes.

The behavioural conditioning literature here overlaps with the management literature about the design of indicators, which is summed up in the use of the acronym SMART. Although the acronym is widely used, there is some disagreement as to what the constituent initials stand for! There is agreement that indicators need to be Specific and Measurable, as discussed above. The A usually stands for Achievable, (sometimes the lexically similar, Attainable) and this is certainly consistent with behavioural approaches. The A may also refer to Agreed, Appropriate, or Action-oriented. Young et al have suggested a key criterion is that providers are aware of the relevant incentives and thus the appropriate indicators [58]. The R can stand for Relevant but may also be Realistic (if A is not Achievable), Rewarding or Results-oriented. T can be for Timely as discussed earlier but it is sometimes Time-bound/Time-based or Tactical.

In line with standard behavioural design principles, the link between the behaviour and the reward needs to be clear and simple enough for hospital/area to be able to understand [62]. Although there may be multiple objectives that the policy maker might wish to achieve, the more complex the formula or the more complex the reward structure, then the effectiveness of the reward or incentive may be undermined and hospitals and areas may not understand what is the intent of the rewards system and not pursue the desired targets. Incorporating performance on multiple dimensions into a single measure involves an inherent loss of transparency and disguises potential tradeoffs and variable performance across disparate measures [63]. There must also be 'valid and comprehensible' management information systems to allow managers to track performance against the goals [64]. Transaction costs and the reporting burden of new indicators should be minimised [45], suggesting that indicators used for rewarding performance in achieving adjunct goals should build on existing (known and validated) information systems, data collections, measures and feed back tools.

A second principle is that the structure of adjunct incentives must be stable and large enough for hospitals to believe that introducing new processes and policies is warranted [28]. The organisation changes necessary to achieve the reward may take more than one year or policy cycle to implement and be disruptive to established organisational routines and hospitals must be able to bring resources to bear to effect the necessary change [62]. If the incentive structure changes on an annual basis (or even more frequently), then hospitals or areas may not feel that there is sufficient stability of policy to warrant the internal costs of re-structure to attempt to gain what might be seen as an ephemeral reward [12,16]. In addition to organisational process changes, there may also be information systems change and other costs involved in documenting that an organisation meets the adjunct incentive criterion. The net expected reward including net reputational benefits [16,40] must at least compensate for these costs and this becomes a "participation constraint" [12]. The literature offers little guidance on what is 'large enough' to induce participation and have an effect on behaviour [37,65], although as indicated above, economic theory indicates that incentives at the margin should have the desired effects.

Thirdly, the design of adjunct incentives needs to be aligned and consistent with all the goals of the payment system design and with policy objectives more broadly. The structure of adjunct incentives needs to be undertaken with some care to ensure that there are no inadvertent perverse incentives such as designing objectives to achieve improved access that can be achieved at the expense of quality goals.

Finally, hospitals are not immune to the temptation to game the system [66-68], and so, as with any payment system, there needs to be an audit trail. Although it is recognised that a move to casemix-based funding systems will lead to improved recording and some 'DRG creep' [69,70], use of patient records and other data in payment system design may also lead to fraud. The more patient record or other data are being used to determine levels of payment, the more there needs to be rigorous audit of these data sources to identify and discourage fraud. Penalties for miscoding need to be stronger than simply rectifying the error.

So what are the key lessons for development of adjunct incentives:

• Be clear and explicit about the behaviour to be rewarded;

• Design incentives so that all facilities with an opportunity to improve have an opportunity to benefit;

• Ensure the reward structure is stable and meaningful;

• Monitor performance and gaming.

## Conclusion

Although the evidence for pay-for-performance is still developing [16,26] and there are still unaddressed research questions about the design of incentive structures [51], the introduction of formula based funding provides the opportunity to funders and purchasers to use price or market-like incentives to achieve a range of policy goals. The more market-like or price incentives are used, the more there is a natural alignment of the incentives of the purchasers/funders and providers. Price and market-like incentives rely on direct and measurable targets that can be translated into price signals, but care must be taken in the design of these market-like incentives. The structure of the incentives must be simple enough to be understood, stable enough to give surety to hospitals that effort in achieving in them is warranted. Subject to these design issues, price and market-like incentives can be used to help improve service provision across a range of domains, not only in terms of technical or allocative efficiency which is often regarded as the only benefit to be achieved from the introduction of formula funding.

## Competing interests

The authors declare that they have no competing interests.

## Pre-publication history

The pre-publication history for this paper can be accessed here:


